# Dental Care Access and the Elderly: What Is the Role of Teledentistry? A Systematic Review

**DOI:** 10.3390/ijerph17239053

**Published:** 2020-12-04

**Authors:** Luca Aquilanti, Andrea Santarelli, Marco Mascitti, Maurizio Procaccini, Giorgio Rappelli

**Affiliations:** 1 Department of Clinical Specialistic and Dental Sciences, Polytechnic University of Marche, 60126 Ancona, Italy; l.aquilanti@pm.univpm.it (L.A.); marcomascitti86@hotmail.it (M.M.); m.procaccini@staff.univpm.it (M.P.); g.rappelli@staff.univpm.it (G.R.); 2 Dentistry Clinic, National Institute of Health and Science of Aging, IRCCS INRCA, 60126 Ancona, Italy

**Keywords:** teledentistry, elderly, residential aged care facility, in-home assistance, public health, dental public health, oral care access

## Abstract

A high level of unmet oral health needs is very common among elderly people. In a society that is getting older and that has been hit so hard by the coronavirus pandemic, the development of new strategies aimed at enhancing general and oral health status should be crucial in order to promote healthy aging. The aim of this systematic review is to assess the feasibility of Teledentistry in communities or in a domiciliary setting where elderly people live. A structured and systematic research was performed on the major electronic databases for studies published in English until 30 June 2020: the PubMed, Cochrane Library, Web of Science, Scopus, and CINAHL databases. A total of 13 articles were identified through database searching using combinations of keywords. Out of 13 papers, eight abstracts were reviewed to assess if they were coherent with the aim of the study, and full texts were retrieved. After abstract reviews, seven articles were selected for closer inspection. Of these, six were assessed for eligibility. Four papers were aimed at assessing patients and health practitioners experiences about Teledentistry, three studies reported a cost analysis and cost description of Teledentistry in residential aged care facilities, and two studies investigated the feasibility and accuracy of Teledentistry for diagnosis dental pathology. The implementation of Teledentistry in residential aged care facilities and in home-assistance programs could be a viable tool for the management of oral care in people who cannot access dental care.

## 1. Introduction

The crisis imposed by the coronavirus (SARS-CoV-2) pandemic has affected public health systems around the world. The rapid spread of SARS-CoV-2 has constricted many countries to restrict human mobility [1]. During the pandemic, dental care has been limited only to urgent and not deferrable treatments. Blood and saliva exposure and droplet production put dental practitioners at high risk of contagion during their routine procedures [2,3,4]. The inhalation of droplets and aerosol from SARS-CoV-2-positive subjects and even direct contact with mucous membranes, oral fluids, and contaminated instruments and surfaces could enhance virus transmission during dental procedures [5,6]. The implementation of fully digital workflows could be useful in order to limit cross-infection [7]. Moreover, this natural pandemic is also responsible for the onset of feelings that may impact the willingness of people to undergo dental appointments [8].

Oral health is part of general health, with a role in the quality of life [9]. In the next few months, health systems will need to treat a large number of patients whose health has been compromised by chronic untreated diseases, including oral diseases. The preservation of oral health is crucial, as is preventing and possibly treating all the diseases that could lead to edentulism, especially in elderly people [10,11,12]. A high level of unmet oral health needs is very common among elderly people, suggesting that enhancing access to dental care is crucial [13]. Oral diseases could be also responsible for the triggering or promotion of inflammatory and infectious processes at a systemic level, potentially worsening the clinical picture of subjects with comorbidities [14].

Inequalities among different social, ethnic, and economic groups highly affect access to and use of dental care services [15]. These inequalities, combined with the current situation imposed by the SARS-CoV-2 pandemic, may become even more critical for elderly people living in long-stay care institutions or with in-home assistance. Elderly people should be isolated to prevent virus infection, but while SARS-CoV-2 infection could be avoided, on the other a worsening of clinical conditions could be observed due to the lack of specialistic check-ups.

The global crisis generated by the pandemic offers a unique opportunity to reshape the traditional approach. Confined and institutionalized elderly people, in particular those with frailty, cannot receive conventional dental care. Reduced mobility and SARS-CoV-2 fear prevent older adults from being treated in a conventional way in dental clinics. In order to improve the quality of care for people with a loss of autonomy and institutionalized care, the use of Teledentistry could be a strategy aimed at providing health for elderly adults during and after the coronavirus pandemic. Dentistry and policymakers must reflect on current and subsequent scenarios, considering the needs and emerging opportunities that will be created after these times.

Teledentistry, defined as the use of health information technology and telecommunications for oral care, was addressed to have the potential to identify high-risk populations; facilitate patient access to dental care; and reduce waiting lists, unnecessary travel, loss of productivity, and also inequalities in dental care access and costs for national health systems [16,17]. In a society that is getting older and that has been hit so hard by the coronavirus pandemic, the development of new strategies aimed at enhancing general and oral health status should be crucial in order to promote healthy aging. The aim of this systematic review is to assess the feasibility of Teledentistry in communities or in a domiciliary setting where elderly people live. In particular, the present review focused on the evaluation of the accuracy and the effectiveness of Teledentistry compared to traditional face-to-face dental visits, the patient acceptability, and the costs related to the implementation of oral health information technology provision.

## 2. Materials and Methods

This systematic review was performed in accordance with the recommendations of the “Preferred reporting items for systematic reviews and meta-analyses protocols (PRISMA-P) statement” [18]. In accordance with the guidelines, the present systematic review protocol was registered in the International Prospective Register of Systematic Reviews (PROSPERO) on 4 September 2020 (registration number CRD42020200827).

### 2.1. Information Sources

Structured and systematic research was performed on the major electronic databases for studies published until 30 June 2020: the PubMed, Cochrane Library, Web of Science, Scopus, and CINAHL databases.

### 2.2. Search Strategy

The following keywords were used in order to perform database searches: “teledentistry”, “elderly”, “aged”, “older”, “elder”, “geriatric”, “nursing homes”, “nursing home”, “long term care”, “residential care”, and “home assistance”, in combination with the Boolean operators “AND” and “OR”. A pilot search was undertaken in order to ensure that the search strategy was effective. The study focused on the Population/Patient, Intervention, Control/Comparison, Outcome(s) (P.I.C.O.S.) criteria [19]. In particular, studies involving elderly people in nursing homes, in communities, or with in-home assisted were included. Subjects requiring oral health care of any sex, ethnicity, socio-economic status, and comorbidities were considered. Studies assessing the use and the applicability of Teledentistry in such structures were included. Any type of oral health provision through the use of health information technology was examined: screening, diagnosis, support, consultation, education, and any other kind of application in dental medicine. Interventions aimed at assessing the above-mentioned interventions provided both using Teledentistry and the traditional face-to-face dental visits were compared. The primary outcome of the review was the comparison between the accuracy and effectiveness of Teledentistry and face-to-face dental visits. The secondary outcomes were the assessment of Teledentistry advantages and disadvantages with the evaluation of patient acceptability and the cost-effectiveness ratio.

### 2.3. Eligibility Criteria

Eligible studies were (a) studies published in the English language; (b) studies published in a peer-reviewed journal; (c) studies published until June 30th, 2020; (d) clinical studies. Studies were excluded if they were: (a) reviews, editorials, commentaries, letters, book chapters, reports on prospective ideas and futuristic scenarios (protocols included), and dissertations.

### 2.4. Data Extraction

Two reviewers (L.A. and M.M.) carried out the evaluations independently. The very first selection was made on the basis of papers title or abstract and eligible ones were selected for full text review. For the assessment of each publication, Excel spreadsheets were compiled. Data were extracted using a standardized form which included (a) authors’ names and the year of publication, (b) country in which the study was performed, (c) the type of study, (d) the setting of the study (e.g., home, community, residential aged care facility), (e) the aim of the study, (f) the type of oral health provision using Teledentistry, (g) the sample size, (h) the mean age ± standard deviation (when applicable), (i) the retrieved article main outcomes, (j) the retrieved article secondary outcomes, (k) the quality assessment score, and (l) the quality of economic evaluation score. Both the authors compared each other and confirmed the data on the basis of the compiled spreadsheets. In case of doubt, concerning the study data, the two reviewers resolved disagreements by discussion. In the case of doubt, a third reviewer solved discrepancies. 

### 2.5. Quality Assessment

The quality of the studies included in the review was evaluated by the two independent reviewers using the protocol described by Hailey et al. [20,21]. This approach provides a quantitative measure of scientific rigor. The overall quality score, assessing both the performance and study design, provides an indication of the degree of confidence of the studies’ findings and their implication for future decision making regarding Teledentistry. Briefly, when reviewing a Telemedicine study, the strength of evidence is defined by the study performance and study design. For the study performance, five different areas of interest are defined: (a) the patient selection, (b) the description/specification of the interventions, (c) the specification and analysis of the study, (d) the patient disposal, and (e) the outcomes reported. For each category, a score of 0, 1, or 2 is given: 0 if relevant information is missing or given in little detail, 1 if reasonable details are provided but with some important limitations, 2 if the information has no significant limitations. For study design, four different scores are assigned: a score of 5 is allocated to large randomized controlled trials (RCTs) with at least 50 subjects in each arm, a score of 3 to smaller RCTs, a score of 2 to prospective not randomized studies, and a score of 1 to retrospective comparative studies. Basing on the totals of the quality scores, each study is assigned to a category that varies from A to E, where “A” indicates studies with the highest degree of confidence and “E” those with the lowest. In particular, categories A, B, C, D, and E correspond to a total quality score of 11.5–15.0, 9.5–11.0, 7.5–9.0, 5.5–7.0, and 1–5.0, respectively.

### 2.6. Quality of Economic Evaluation

The quality assessment of the studies that included a cost analysis was performed in accordance with the Drummond et al. 10-point checklist [22]. For each eligible paper, a score was assigned for each of the following criteria:

1. Was a well-defined question posed in answerable form?
2. Was a comprehensive description of the competing alternatives given?
3. Was the effectiveness of the programs or services established?
4. Were all the important and relevant costs and accurately in appropriate physical units?
5. Were the costs and consequences measured accurately in appropriate physical units?
6. Were the costs and consequences valued credibly?
7. Were the costs and consequences adjusted for different timing?
8. Was an incremental analysis of costs and consequences of alternatives performed?
9. Were allowances made for uncertainty in the estimates of costs and consequences?
10. Did the presentation and discussion of the study results include all issues of concern to users?

Each item had three possible answers: “yes”, “cannot tell”, and “no”. The response score was 1.00, 0.50, and 0, respectively. A 0.5 value was set for the “cannot tell” answer because the absence of any information has an equal probability of either a “no” (score = 0) or a “yes” (score = 1). The lowest and highest possible scores were 0 and 10. A total score of 5.00 was considered the minimum threshold score to consider an economic evaluation of sufficient quality and suitable for the inclusion in an economic evaluation database.

## 3. Results

**Figure 1:**
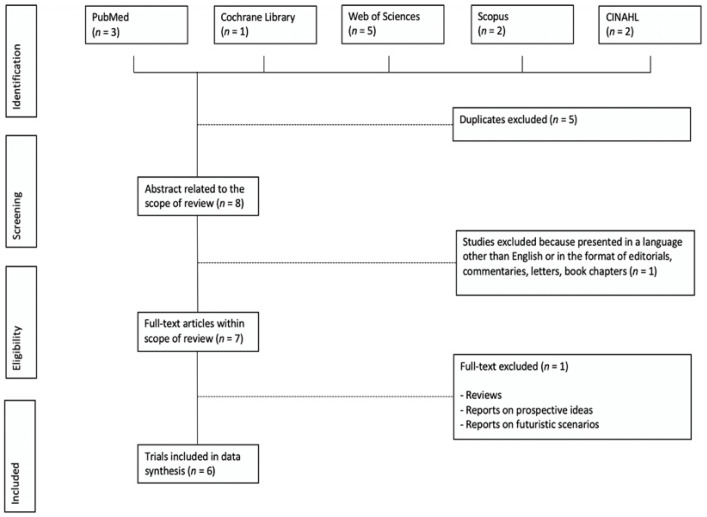
The preferred reporting items for systematic reviews and meta-analysis (PRISMA) flowchart of the search results and selected studies.

A total of 13 articles were identified through database searching using combinations of keywords. Out of 13 papers, eight abstracts were reviewed to assess if they were coherent with the aim of the study and full texts were retrieved. Five studies were excluded because they were duplicates. After abstract reviews, seven articles were selected for closer inspection. A study was not included because it considered young patients. Of these seven articles, six were assessed for eligibility, while a study was excluded because it did not meet the inclusion criteria (Figure 1).

The studies included in the present review were conducted in three different nations: Australia (*n* = 4), France (*n* = 2), and Germany (*n* = 1). A high heterogeneity was assessed among the retrieved articles, reporting different outcomes. Two studies investigated the feasibility and the accuracy of Teledentistry for diagnosis dental pathology using the traditional face-to-face examination as gold standard [23,24]. Four papers were aimed at assessing patients and health practitioners experiences about Teledentistry by the use of questionnaires [23], direct observation of nurses [25], surveys and clinical audit charts [26,27]. Finally, three studies reported also a cost analysis and a cost description of Teledentistry in residential aged care facilities [23,26,28].

Full-text articles that met the eligibility criteria are included in Table 1. Table 1 shows (a) the authors’ names and year of publication, (b) the country in which the study was performed, (c) the type of study, (d) the setting of the study, (e) the aim of the study, (f) the type of oral health provision using Teledentistry, (g) the sample size, (h) the mean age ± standard deviation (when applicable), (i) the retrieved article main outcomes, and (j) the retrieved article secondary outcomes.

Regarding the assessment of the feasibility of Teledentistry in screening oral diseases and conditions and in developing treatment plans, Mariño et al. tested both virtual and traditional oral examination [23]. The intra-examiner agreements, determined by the Kappa index, indicated an excellent agreement, Kappa = 0.83. Queyroux et al. compared the accuracy of Teledentistry approach to the conventional face-to-face oral examination [24]. Diagnoses were classified as true positives, as false negatives and false positives and sensitivity, specificity, positive predictive value and negative predictive value were calculated. More receiver operating characteristic analyses were performed in order to determine the diagnostic performance of Teledentistry. Virtual examination lasted 12 min, while the traditional visit took 20 min. The results of the diagnostic accuracy for dental pathology showed a sensitivity of Teledentistry of 93.8% (95% CI: 90.7–96.9%), a specificity of 94.2% (95% CI: 91.2–97.2%), a positive predictive value of 95.2% (95% CI: 92.4–98.0%), and a negative predictive value of 92.4% (95% CI: 89–95.9%). In the receiver operating characteristic analysis, the area under the curve was 0.95 (95% CI: 0.92–0.98). The assessment of accuracy for chewing ability indicated that the sensitivity of Teledentistry was 85.0% (95% CI: 80.0–90.0%), the specificity 82.8% (95% CI: 77.7–88.1%), the positive predictive value 92.2% (95% CI: 88.5–95.9%), and the negative predictive value 69.6% (95% CI: 63.2–76%). Sensitivity, specificity, positive predictive value, and negative predictive value for the assessment of accuracy in the evaluation of dental prostheses rehabilitation status were 87.8% (95% CI: 82.5–93.1%), 90.3% (95% CI: 85.5–95.1%), 78.3% (95% CI: 71.6–85.0%), and 94.9% (95% CI: 91.3–98.5%), respectively. Overall, Teledentistry had excellent sensitivity and specificity for diagnosing dental diseases among elderly people living in residential aged care facilities.

Among the positive aspects that this kind of technology could add to the provision of oral care in residentials, we found: (a) the absence of adverse effects, (b) the reduction in waiting lists and unnecessary travels, and (c) the minimization of disruption to high-care residents, particularly those diagnosed with dementia. Moreover, the use of Teledentistry could be a useful tool also for the improvement of oral care education among residential aged care facilities workers, patients and residentials families. Overall, participants experience about Teledentistry was assessed using questionnaires, direct observation of patient behavior, chart audits and interviews. Only, two papers reported negative experiences among the participants of the studies [23,25]. Fear and anxiety were the two main feelings generated by Teledentistry in patients together with a dispositional resistance, not to the use of technology, but to the whole dental procedure [25]. According to Mariño et al., controversy than residents, although understanding the opportunities given by technology, the nurses of residential aged care facilities questioned Teledentistry effectiveness, claiming that the proposed method was not capable of recognizing the reality of residential aged care facilities [23]. Nevertheless, when participants were asked to rate their experience with Teledentistry, almost the totality of them were either “very satisfied” or “slightly satisfied” (46% and 38%, respectively), strongly or slightly recommending the remote dental examination to other people of their age (46% and 46%, respectively). Moreover, the participants considered remote communications generally either “very easy” or “easy” to understand, at 46% and 46% respectively. Only 4% of the participants found it “difficult” or “very difficult”, complaining about the foreign accent of oral health professional rather than the technology used. The 28% of the residents asserted that the most valuable element of Teledentistry was its convenience in terms of costs savings and disruption and difficulty avoidance [23]. Finally, Queyroux et al. reported that Teledentistry was not associated with any severe negative effect and an excellent acceptability rate was recorded among both residents and their families (95.3%) [24]. A positive feedback was also evaluated among residential staff, residents and their families by Tynan et al. [26].

### 3.1. Quality Assessment in Individual Studies

The quality of the studies included in the review was evaluated using the protocol described by Hailey et al. [20,21]. The available literature constricted to include in the present review mostly articles with poor or poor to fair quality, characterized by substantial limitations in the study [23,25,26,27,28], and only one with fair to good quality [24].

### 3.2. Quality of Economic Evaluation

Overall, three studies reported an economic evaluation of Teledentistry and scored five or more according to the criteria suggested by Drummond et al. [22]. In particular, Mariño et al. [28] was given a total score of 7, meeting 5 out of the 10 criteria and receiving a 0.5 value for 4 of them. Mariño et al. [23] scored 6, fulfilling 4 out of the 10 conditions and getting a 0.5 score for 4 of them. Finally, Tynan et al. [26] met 3 out of the 10 points and receiving a score of 0.5 for 4 of them, totally scoring 5.

The economic analyses in the reviewed articles showed that the studies were cost-analysis ones. All the pertinent costs from a healthcare perspective were identified and calculated using market values, published official salaries, and expert opinions. Generally, three main categories of costs were defined: (a) training, (b) salaries, and (c) teledental device [23]. Mariño et al. estimated the unit cost of Teledental consultation, considering both the real-time method and the storage and forward [23]. The cost of training was equal between the two options, while the costs related to intervention provision were different. A higher amount of time was registered in the storage and forward method (20 min vs. 15 min for the real-time oral examination), thus slightly augmenting, but not significantly, the cost of this Teledentistry option, comparing it to the real-time model. Mariño et al. compared costs per treatment plan development among face-to-face oral examination performed by an oral health professional, storage and forward Teledentistry model and real-time Teledentistry model [28]. The total cost of storage and forward model was fewer than the real-time one, representing the cheapest option. When considering the conventional face-to-face oral examination, additional costs for dental assistant and travel should be added to the amount. Overall, the storage and forward option is the always the cheapest alternative, when comparing it to both the real-time method and the conventional oral examination. The asynchronous model was cheaper than the real-time one because of personnel costs, while for the traditional approach costs increased due to travel for oral professionals and dental assistants. Tynan et al. analyzed the costs generated by the implementation of an oral health therapist in residential aged care facilities, the utilization of a Teledentistry model and the attendance at oral health clinic by resident (via car or ambulance) [26]. The screening performed by the oral health therapist was shown to be the lowest cost model, while the costs were augmented when considering the Teledentistry model due to the cost of both set-up and dentist time, and residents’ attendance at a dental clinic incurred the highest costs due to the cost of transportation. The disruption of high-care patients could be also taken into account.

### 3.3. Data Synthesis for Meta-Analysis

A meta-analysis was not performed in order to avoid the systematic error that could have occurred due to the selection and publication bias and the heterogeneity among the studies. Therefore, neither I^2^ nor the Cochran Q value are reported in the present systematic review.

## 4. Discussion

The present review identified literature regarding the application of Teledentistry in geriatric settings. Overall, there is a strong trend supporting the feasibility of Teledentistry compared to the traditional approaches [29]. If on one hand, most of papers stated that Teledentistry is comparable to or even better than the conventional alternative, on the other, conclusive statements are not possible to be drawn and publication bias could be met. Only few studies are well constructed and reported a controlled comparison between Teledentistry application and face-to-face alternatives [30]. Regarding the application of Teledentistry in the management of elderly people living in residential aged care facilities, available literature is even fewer. Papers concerning older people at in-home assistance are null. Only two studies reporting oral home telecare for adults with tetraplegia are available, showing that oral home telecare offers the opportunity to decrease physical barriers and to improve the quality of dental health services for people at in-home assistance [31,32].

Teledentistry belongs to the natural process of the digitalization of modern society and medicine. It is able to improve services, breaking down barriers and allowing also people with limited or no possibility to access to dental care [33,34]. The relationship between health and inequalities has been deeply investigated by considering different professional and social conditions, showing that mortality rates increase in proportion to economic and social hardship, lower incomes, education, and social class [35]. Oral health problems are more prevalent in lower social strata, so the access to dental health services has deteriorated in Europe over the years, especially in countries severely hit by the economic crisis, such as Greece, Spain, Portugal, Italy, and Ireland [36]. Beside economic crisis, oral health access could be restricted by the bad clinical conditions of the subjects. In the USA and Australia, the number of elderly people living in rest homes have dramatically risen [37,38]. It was estimated that the 65% almost of who used to live in nursing homes suffered from oral diseases. Moreover, poor oral status is a strong predictor of the onset of adverse health outcomes, including mortality among the community-dwelling elderly [39,40]. The individualization and the assessment of factors associated with such conditions will help in the prevention or minimization of their negative consequences on health. The implementation of Teledentistry in residential aged care facilities could assists users to access oral care, providing regular visits using trained caregivers in the first instance.

Overall, the present systematic review identified three main topics, consistent with the main and the secondary outcomes of the present review: (a) accuracy and effectiveness, (b) acceptability, and (c) costs. Teledentistry was found to have an excellent accuracy for the diagnosis of dental diseases and good accuracy for the assessment of chewing ability and oral rehabilitation status among elderly people living in nursing homes. Despite the different aims, methods, and outcomes of the studies, virtual dental examination was comparable to the traditional face-to-face dental visit [23,24]. If on one hand, Teledentistry could provide general and specialist oral health care support to elderly people, on the other the implementation of Teledental assistance should not substitute the traditional approach in the case of the suspect of more severe diseases that need in-depth diagnostic procedures. As oral health is considered to be an indicator of frailty and the improvement of oral conditions among elderly people is needed, Teledentistry could be used for screening and preventing dental pathologies in older people [24,41]. Moreover, a previous study stated that Teledentistry has the capacity to reduce appointment waiting lists, triaging and prioritizing appointments, and to provide a rationalization of time, travel and costs for all parties [42]. Additionally, teleconsultation could reduce all the stress generated by travelling to a dental office from home or the residential aged care facility of the patients, especially in those with dementia that could lead to a complete lack of collaboration with dental practitioners [27].

Overall, a high level of acceptability of Teledentistry was reported in the majority of the articles included in this review among patients, patients’ families, and caregivers. Survey-based studies showed satisfaction among the users because of the increased confidence in residential aged care facilities [23]. Positive feedback was received also because of the augmented awareness of oral health needs and management, savings in residents transportation to oral care facilities, and positive cultural change among caregivers through remote education [26]. Moreover, the audits showed an improved compliance and consciousness of the importance of oral health in facilities with access to dental consultation [27]. Conversely, Petcu et al. reported that psychotic patients perceived Teledentistry more negatively than nonpsychotic ones [25]. Among nonpsychotic patients, the total negative experience became more distinct moving from dependent patients to semi-autonomous and to autonomous ones. Fear and anxiety were the main feelings generated by the procedure as a whole and not by the use of technology. The latter, as suggested by a recent review, could have an added benefit in the provision of oral care, particularly in odontophobic subjects [43]. Other negative feedback could be addressed to the lack of immediate response on the examination and the resistance from some nurses who questioned the effectiveness of Teledentistry in the context of residential aged care facilities [23]. While, for the first point, the use of a real-time Teledentistry model could solve the problem of immediate feedback, for the second one, the skepticism may be due to the absence of innovative oral care programs in most nursing homes.

When comparing Teledentistry to face-to-face oral examinations, a reduction in costs was likely to be detected [44]. A study with a micro-costing analysis and direct cost measurement was performed from a healthcare perspective, trying to find out the cost of implementing an asynchronous and real-time Teledentistry model in a nursing home setting: no significant differences were detected between the two methods [23]. Conversely, in a model cost-analysis format, Mariño et al. compared the traditional examinations to the storage and forward Teledentistry method and the real-time one [28]. The authors stated that the asynchronous model was always the less costly one, followed by the real-time model and face-to-face dental visits. Tynan et al., in turn, estimated the costs of three different scenarios, including the implementation of an oral health therapist in the residential aged care facility, the use of Teledentistry, and patient transportation to a dental facility [26]. The first option was considered to be the least-cost scenario, followed by the use of Teledentistry and the third option. Nevertheless, in the latter study, an oral health therapist was supposed to perform the intraoral acquisition and to transmit it to a dentist placed elsewhere. In addition to the cost of Teledentistry set-up, in this case also the costs of both the oral health therapist and dentist were calculated. Regarding costs, the travel cost also should be considered. The application of Teledentistry in the provision of the oral care of elderly people could allow savings in terms of travel (via car or ambulance), caregiver escort time to accompany the patient, and patient disruption. The additional cost of Teledentistry is associated with training, increasing the total cost amount. However, new skill development among caregivers and the strengthening of health team capacities could be achieved through supportive environments and remote learning sessions [42].

## 5. Conclusions

The present systematic review is the first that analyzed the feasibility of Teledentistry in the provision of oral care in elderly people living in residential aged care facilities or with in-home assistance. In a society that is getting older, the implementation of Teledentistry in residential aged care facilities and in-home assistance programs could be a viable tool for the management of oral care in people who cannot access dental care. Moreover, in a context in which movement is not recommended for the most vulnerable categories, avoiding unnecessary appointments and triaging dental visits could be effective. Although the absence of high-quality studies limited the findings, Teledentistry was found to be as accurate as traditional face-to-face dental examinations; cost-effective; and well accepted among patients, patients’ families, and caregivers. Well-designed studies aimed at assessing Teledentistry and surroundings are therefore needed in order to increase the body of evidence supporting the feasibility of the digitalization of oral care.

## Figures and Tables

**Table 1:** Summary of the studies included in the review.

Authors and Year	Country	Type	Setting	Aim	Application	Sample Size (*n*)	Age	Main Outcome	Secondary Outcome
Mariño et al. [23]	Austra2lia	Pilot feasibility study and cost-analysis	Residential aged care facilities	To assess Teledentistry safety, feasibility, and acceptance	Development of treatment plans	50	N/A	High concordance between remote and face-to-face examination Patient satisfaction with Teledentistry	Provision of regular and timely oral health checks Travel-associated stress reduction Prioritization of appointments Improvement of efficacy and increment of number of visited residents Improvement of confidence in resident aged care facilities Implementation barriers largely due to human factors
Queyroux et al. [24]	France and Germany	2-year, multicenter, cross-sectional study	Nursing homes	To assess Teledentistry accuracy using direct examination as gold standard	Dental pathology	237	84.4 ± 8.3 years	Dental pathology Sensitivity: 93.8% Specificity: 94.2% PPV ^1^: 95.2% NPV ^2^: 92.4%	Chewing ability Sensibility: 85.0% Specificity: 82.8% PPV ^1^: 92.2% NPV ^2^:69.6% Dental prostheses status Sensibility: 87.8% Specificity: 90.3% PPV ^1^: 78.3% NPV ^2^: 94.9% Use of Teledentistry Not associated with any serious adverse events Excellent acceptability rate (95.3%) Quicker than face-to-face examination (12 vs. 20 min, respectively)
Petcu et al. [25]	France	Report based on the preliminary result of a project	Long term facilities for the elderly and specialized facilities for adults with severe intellectual, motor or somatic disabilities	To evaluate the acceptability of Teledentistry among elderly people	Screening	123	60% of the sample were 65 years old or older	Onset of anxiety regarding the procedure Onset of dispositional resistance	N/A
Tynan et al. [26]	Australia	6-months, quality improvement study and cost-analysis	Residential aged care facilities	To describe the development and implementation of Teledentistry model	Screening	116	N/A	Improvement in implementation of oral care plans Minimization of need to attend an oral health care facility	No adverse events Costs minimization Positive feedback from staff, residents and families Minimization of disruption to high-care residents (particularly those with dementia)
Tynan et al. [27]	Australia	Mixed methods comparative study	Residential aged care facilities and multi-purpose health services	To investigate the impact and the experience of Teledentistry compared to traditional approaches in residential aged care facilities	Screening	252	79.8 years	Improving oral health education among staff and residents Minimization of disruption to residents (especially those with dementia)	Reduction in inequities in dental care access Improvement of oral care education, promotion, oral diseases prevention and timely intervention Potential reduction in waiting lists and unnecessary travel Familiar setting
Mariño et al. [28]	Australia	Cost-analysis comparison study	Public healthcare	To compare the costs of face-to-face examination with two different Teledentistry approaches in residential aged care facilities	Screening	N/A	N/A	Asynchronous Teledentistry model has the lowest cost compared to both real-time Teledentistry and face-to-face examination	Caregivers training and education

^1^ Positive Predictive Value: true positives/(true positives + false positives); ^2^ Negative Predictive Value: true negatives/(true negatives + false negatives).
